# Feasibility of quantitative ultrasonography for the detection of metabolic bone disease in preterm infants — systematic review

**DOI:** 10.1007/s00247-018-4161-5

**Published:** 2018-06-16

**Authors:** Liting Tong, Jaya Sujatha Gopal-Kothandapani, Amaka C. Offiah

**Affiliations:** 1 Mid Yorkshire Hospitals NHS Trust, Wakefield, UK; 2 Department of Oncology and Metabolism, University of Sheffield, Sheffield, UK; 3 Academic Unit of Child Health, Damer Street Building, Sheffield Children’s NHS Foundation Trust, Western Bank, Sheffield, S10 2TH UK

**Keywords:** Bone mineral density, Children, Metabolic bone disease, Preterm infants, Quantitative ultrasonography, Review, Speed of sound, Ultrasound

## Abstract

Metabolic bone disease of prematurity is characterised by disordered bone mineralisation and is therefore an increased fracture risk. Preterm infants are especially at risk due to incomplete in utero bone accretion during the last trimester. Currently, diagnosing metabolic bone disease mainly relies on biochemistry and radiographs. Dual-energy x-ray absorptiometry and quantitative ultrasound (US) are used less frequently. However, biochemical measurements correlate poorly with bone mineralisation and although scoring systems exist for metabolic bone disease, radiographs are subjective and do not detect early features of osteopenia. Dual energy x-ray absorptiometry is the reference standard for determining bone density in older children and adults. However, challenges with this method include movement artefact, difficulty scanning small and sick infants and a lack of normative data for young children. Quantitative US has a relatively low cost, is radiation-free and portable, and may hence be suitable for assessing bone status in preterm infants. This review aims to provide an overview of the use of quantitative US in detecting metabolic bone disease in preterm infants.

**Electronic supplementary material:**

The online version of this article (10.1007/s00247-018-4161-5) contains supplementary material, which is available to authorized users.

## Introduction

Metabolic bone disease and osteogenesis imperfecta are the two most common causes of fragile bones in infancy [1]. Metabolic bone disease is characterised by skeletal demineralisation and fractures that can occur during normal handling [2]. The in utero process of bone accretion increases exponentially during the last trimester of pregnancy [3]. Preterm infants are, therefore, deprived of this period of mineral accumulation, have low skeletal mineral stores and are predisposed to developing metabolic bone disease [4].

Other factors that increase their risk of metabolic bone disease include comorbidity, immobility and the use of drugs such as steroids and loop diuretics [3]. Concurrent use of total parenteral nutrition with an inadequate mineral content to match the infant’s higher metabolic demand leads to abnormal bone remodeling and metabolic bone disease [2, 4].

In a recent study, 30.9% of extremely low birth weight infants had radiologic evidence of metabolic bone disease [5]. In the short term, metabolic bone disease may impair the infant’s respiratory status and may be a factor in the development of myopia of prematurity associated with impaired growth of the skull [4]. These infants are also more at risk of fractures beyond the neonatal period, especially during the first 2 years of life [6]. In the same study, about a third of infants with metabolic bone disease developed spontaneous bone fractures [5].

In adolescence, former preterm infants tend to be shorter and lighter for their age and have been reported to have lower bone mass, bone mineral content, bone density and cortical cross-sectional area [4, 7, 8]. Despite the use of mineral-enriched preterm formulas, advances in intensive neonatal care and a reduction in the use of steroids and diuretics, metabolic bone disease remains a significant comorbidity. It has been reported that the incidence of metabolic bone disease in very low birth weight infants and extremely low birth weight infants is 32% and 54%, respectively, and that 10% of very low birth weight infants may be at risk for fractures [9, 10].

Considering these short- and long-term complications of poor neonatal bone health and the increasing survival rates for very low and extremely low birth weight preterm infants, an improved method of assessing bone health is necessary.

## Current assessment of bone health

Currently, metabolic bone disease diagnosis relies on biochemical evaluation and radiologic investigation [3]. Biochemical measurements include serum or urinary phosphate, serum calcium and alkaline phosphatase [4]. A raised alkaline phosphatase and low serum phosphate may indicate metabolic bone disease. However, biochemical features correlate poorly with bone mineralisation and may not be consistent indicators of bone strength or mineralisation [6]. Conventional radiographs may be used to look for osteopenia or fractures and to grade metabolic bone disease [10]. However, radiographs are poor at diagnosing mild bone disease and radiologic features of osteopenia only become reproducibly apparent after 30–40% of mineral loss [2, 4].

Dual energy x-ray absorptiometry (DXA) is used to determine bone mineral density, which correlates with bone mineralisation and bone mineral content. DXA is the gold standard in adults and children. However, the lack of portable machines and the small size of (preterm) neonates and infants (who may be very ill) pose challenges for its use [4]. Furthermore, data from DXA scans are difficult to interpret in newborns due to movement artefact and variations in technique [4]. Overall, it is also relatively expensive [7]. Another important limitation of DXA is that it measures bone in just two dimensions, thus only providing an estimate of bone mineral density, which in children is highly variable because of changes in bone geometry with growth. Scientists have not agreed on a mathematical formula to fully account for differences in bone size [11].

The main advantages of DXA are its wide availability, short scanning times and low radiation dose [11].

Assessing bone health and/or diagnosing metabolic bone disease in the preterm infant remains difficult as there is no screening test that is both specific and sensitive. Biochemical indices are not diagnostic, radiographs have low sensitivity, and DXA is impractical for routine use and of questionable reliability [4].

## Quantitative ultrasonography

Quantitative ultrasonography (US) was developed in 1984 as a non-ionising, portable and low-cost alternative to conventional methods of measuring bone health [4]. Quantitative US follows the principle that velocity of transmission and amplitude are influenced when a US wave is propagated through bone [11]. Many quantitative US devices are specific to only one skeletal site, such as the calcaneum or tibia. A US transducer and receiver are placed at opposite ends of the bone. The US wave passes through the area of interest and parameters such as speed of sound (speed of propagation of US wave through bone) and bone transmission time (time taken for ultrasonic wave to pass through bone) are recorded [4]. Speed of sound increases and bone transmission time decreases with an increase in bone density and strength. The parameters reflect bone density, architecture and elasticity, including qualitative bone properties such as bone mineralisation and quantitative properties such as cortical thickness, elasticity and microarchitecture, providing a more complete picture of bone health as compared to current assessment techniques [4, 11]. This is useful in preterm infants because qualitative bone properties may be affected in addition to bone mineral density, further predisposing them to metabolic bone disease [3].

Quantitative US techniques can be applied to peripheral sites, are safe, easy to use and cost effective; the devices are portable and only a few minutes are needed to perform the measurements at the bedside. These characteristics make it favourable for use in assessing bone status in children [11].

In vitro studies have shown that forearm quantitative US variables correlate significantly with bone strength, and these parameters have been found to correspond to bone mineral assessment by DXA in children [7]. Results have demonstrated that quantitative US devices adapted for children can be used as frequently as DXA to estimate bone mineral status and bone fragility, but current data are not sufficient to establish which of them is the best choice [11]. This review will evaluate the potential of quantitative US as an important tool in the diagnosis, management and follow-up of metabolic bone disease in preterm infants. In this review, we evaluate studies that have used a total of four commercially available quantitative US devices: Omnisense 7000P (Sunlight Medical Inc., Tel Aviv, Israel), DBM Sonic (IGEA, Capri, Italy), DBM Bone Profiler (IGEA, Capri, Italy) and Osteoson KIV (Minhorst, Medut, Germany).

## Search strategy

**Fig. 1:**
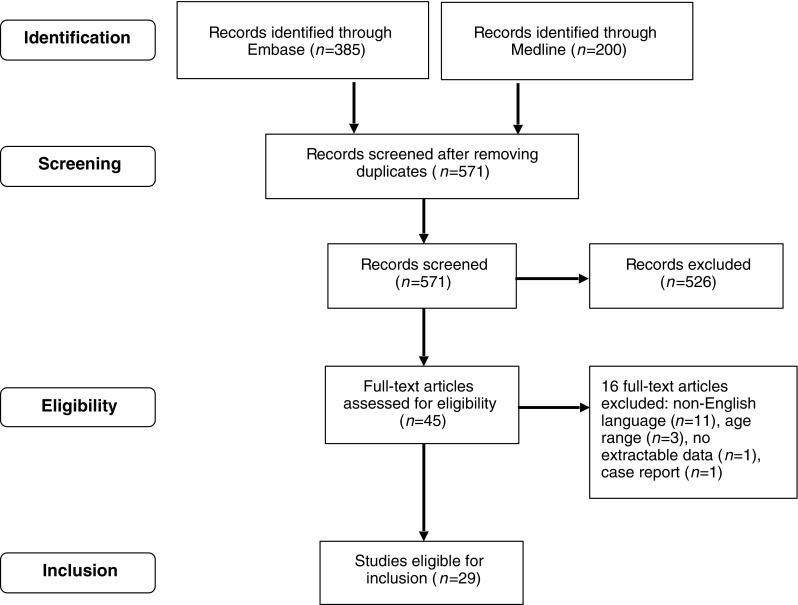
Identification and inclusion of articles for analysis

For literature analysis we used the Critical Appraisal Skills Programme tool [12]. A systematic search (Fig. 1) was performed of Medline and Embase (Table 1). Reference lists from identified studies were hand-searched to identify further relevant studies. No time limits were applied. Unpublished data such as conference proceedings were not included. Articles not written in English were excluded. Twenty-nine papers were included and are summarised in Table 1. The Critical Appraisal Skills Programme tool [12] was also used to assess the quality of these papers and is shown in Table 2.

**Table 1:** Summary of papers included in review

Reference	Year	Quantitative ultrasound device	Site/parameter	Term/ preterm	Study design	*n*	Speed of sound (term)	Age at scan (term)	Preterm speed of sound values	Age at scan (preterm)
Mercy et al. [2]	2007	Omnisense	Tibia/ SOS	No/Yes	Longitudinal	84				5 (2–9)^b^ (days)
Ashmeade et al. [7]	2007	Omnisense	Tibia/ SOS	Yes/Yes	Cross-sectional/ longitudinal	108	3,036 (2,843–3,333)^b^	≤72 h of life	2,924 (2,672–3,220)^b^	≤1 week of life
McDevitt et al. [8]	2007	Omnisense	Tibia/ SOS	No/Yes	Cross-sectional/ longitudinal	39			2,942 (2,609–3,064)^b^ (corrected gestational age 0–6 months) 3,269 (3,009–3,413)^b^ (corrected gestational age 6–12 months) 3,327 (3,110–3,495)^b^ (corrected gestational age ≥ 12 months)	32 (2–104)^b^ (days)
Zuccotti et al. [13]	2011	Omnisense	Tibia/SOS	Yes/No	Cross-sectional/ Longitudinal	116	2,964 (2,811–3,282)^b^ (girls) 3,042 (2,656–3,349)^b^ (boys)	<9 days		
Tansug et al. [14]	2011	Omnisense	Tibia/ SOS	Yes/Yes	Longitudinal	126	3,114 (139)^a^	10th day	2,995 (143)^a^	10th day
Gonnelli et al. [15]	2004	DBM Bone profiler	Humerus/ BTT, SOS	Yes/No	Cross-sectional	140	1,724.8 (25.3)^a^	<3 days		
Betto et al. [16]	2014	DBM Sonic	Metacarpal/ BTT, SOS	No/Yes	Cross-sectional/ Longitudinal	154			1,642.17 (28.35)^a^	<24 h of birth
Ritschl et al. [17]	2005	DBM Sonic	Second metacarpus/BTT, SOS	Yes/Yes	Cross-sectional/ Longitudinal	338	1,684 (27)^a^	<24 h	1,636 (17)^a^	<24 h
Litmanovitz et al. [18]	2007	Omnisense	Tibia/ SOS	No/Yes	Interventional	16				≤7 days
Liao et al. [19]	2005	Omnisense	Tibia/SOS	Yes/Yes	Cross-sectional	542	2,984 (116)^a^	<3 months	2,935 (96)^a^	<3 months
McDevitt et al. [20]	2005	Omnisense	Tibia, distal third of radius/ SOS	Yes/Yes	Cross-sectional	110	3,079 (3,010–3,142)^b^	3 (2–5)^b^ (days)	2,994 (2,917–3,043)^b^ (gestational age 32–36 weeks) 2,911 (2,816–2,982)^b^ (gestational age <32 weeks)	3 (2–5)^b^ (days)
Altuncu et al. [21]	2007	Omnisense	Tibia/SOS	Yes/Yes	Cross-sectional/ Longitudinal	55	z-score: 0.0 ([−0.8]-0.5)^b^	<1 week	z-score: 0.4 ([−0.2]-1.4)^b^	<1 week and term-corrected age
Chen et al. [22]	2012	Omnisense	Tibia/ SOS	Yes/Yes	Cross-sectional	667	2,971.7 (1,06.3)^a^	≤7 days	2,932.9 (112.4)^a^	≤7 days
Rack et al. [23]	2012	Osteoson KIV	4 different sites/ SOS	Yes/Yes	Longitudinal	172	1,785 (27)^a^	≤7 days	1,720 (24)^a^	≤7 days
Littner et al. [24]	2004	Omnisense	Tibia/SOS	Yes/No	Cross-sectional	25	3,082.4 (93.7)^a^	<96 h of life		
Fewtrell et al. [25]	2008	Omnisense	Tibia/ SOS	No/Yes	Cross-sectional/ longitudinal	99			2,950 (2,821–3,220)^b^	2.6 (2.6)^a^ (weeks)
Chen et al. [26]	2010	Omnisense	Tibia/ SOS	No/Yes	Interventional	16			2,851.5 (89)a	At birth
Litmanovitz et al. [29]	2003	Omnisense	Tibia/SOS	No/Yes	Interventional	24			2,892.3 (29.5)^a^ (Control] 2,825.0 (32.2)^a^ [Intervention]	<1 week
Pereda et al. [30]	2003	Omnisense	Tibia/SOS	No/Yes	Cross-sectional	95			No numerical data	2.7 (1.9)^a^ [days]
Littner et al. [31]	2003	Omnisense	Tibia/SOS	Yes/Yes	Cross-sectional	73	No numerical data	<96 h of life	No numerical data	<96 h of life
Rubinacci et al. [32]	2003	DBM Sonic	Humerus/BTT, SOS	Yes/Yes	Cross-sectional	94	1,734 (28)^a^	<1 week	1,664 (42)^a^	At least 34 weeks post conceptual age
Littner et al. [33]	2004	Omnisense	Tibia/SOS	Yes/Yes	Cross-sectional	50	3,010 (118)^a^ (no specific data based on gestation)	<96 h of life	3,010 (118)^a^ (no specific data based on gestation)	<96 h of life
Littner et al. [34]	2005	Omnisense	Tibia/SOS	Yes/Yes	Cross-sectional	22	3,063 (126)^a^ (mean gestation: 34 weeks)	<96 h of life	3,063 (126)^a^ (mean gestation: 34 weeks)	<96 h of life
Teitelbaum et al. [35]	2006	Omnisense	Tibia/SOS	Yes/Yes	Cross-sectional	235	3,012 (98)^a^	<96 h of life	2,963 (132)^a^	<96 hs of life
Chen et al. [36]	2007	Omnisense 7000P	Tibia/SOS	No/Yes	Cross-sectional	144			3,098 (135)^a^ (small for gestational age infants) 3,003 (122)^a^ (appropriate for gestational age infants)	<1 week of life
Ahmad et al. [37]	2010	Omnisense 7000P	Tibia/SOS	Yes/Yes	Cross-sectional	102	3,168.4 (3,129.0–3,207.9)^b^	<3 months	2,797.4 (2,720.4–2,874.4)^b^ (23–28 weeks) 3,003.9 (2,949.8–3,058)^b^ (29–32 weeks) 2,470 (2,267.2–2,673.4)^b^ (33–36 weeks)	<3 months
Liao et al. [38]	2010	Omnisense 7000P	Tibia/ SOS	Yes/Yes	Longitudinal	267	2,979 (113)^a^	≤6 days of delivery	2,945 (89)^a^	≤6 days of delivery
Savino et al. [39]	2013	DBM sonic	Metacarpal/ BTT, SOS	Yes/No	Cross-sectional	103	1,640 (26)^a^	127 (81)^a^ (days)		
Erdem et al. [40]	2015	Omnisense 7000P	Tibia/SOS	No/Yes	Interventional	28			2,901.28 (120.08)^a^ (control) 2,812.0 (149.69)^a^ (Intervention)	Unknown

*BTT* bone transmission time, *SOS* speed of sound

^a^mean (standard deviation), ^b^median (range)

**Table 2:** Application of the Critical Appraisal Skills Programme tool [12]

Quantitative ultrasound device	Study	Year	Type of study	Are the results of the study valid?	What are the results?	Will the results help locally?
Omnisense 7000P	Mercy et al. [2]	2007	Cohort	+	+	±
	Ashmeade et al. [7]	2007	Case control	±	±	±
	McDevitt et al. [8]	2007	Cohort	+	+	±
	Zuccotti et al. [13]	2011	Cohort	±	+	±
	Tansug et al. [14]	2011	Case control	±	+	±
	Litmanovitz et al. [18]	2007	Randomised controlled trial	±	+	±
	Liao et al. [19]	2005	Case control	±	+	–
	McDevitt et al. [20]	2005	Cohort	±	+	±
	Altuncu et al. [21]	2007	Diagnostic accuracy	±	±	±
	Chen et al. [22]	2012	Case control	±	+	±
	Littner et al. [24]	2004	Case control	±	±	±
	Fewtrell et al. [25]	2008	Cohort	±	±	±
	Chen et al. [26]	2010	Randomised controlled trial	±	+	±
	Litmanovitz et al. [29]	2003	Randomised controlled trial	+	+	±
	Pereda et al. [30]	2003	Cohort	±	+	±
	Littner et al. [31]	2003	Cohort	±	±	±
	Littner et al. [33]	2004	Case control	±	±	±
	Littner et al. [34]	2005	Case control	±	±	±
	Teitelbaum et al. [35]	2006	Case control	±	±	±
	Chen et al. [38]	2007	Case control	±	+	±
	Ahmad et al. [37]	2010	Case control	±	±	±
	Liao et al. [38]	2010	Case control	–	±	±
	Erdem et al. [40]	2015	Randomised controlled trial	±	+	±
DBM Sonic	Gonnelli et al. [15]	2004	Cohort	±	+	±
	Betto et al. [16]	2014	Cohort	±	+	±
	Ritschl et al. [17]	2005	Cohort	±	+	±
	Rubinacci et al. [32]	2003	Case control	±	+	±
	Savino et al. [39]	2013	Cohort	±	+	±
Osteon KIV	Rack et al. [23]	2012	Case control	–	+	±

*+* Yes

*-* No

*±* Unable to tell

## Analysis

### Feasibility

Twenty-eight studies reported successful scanning of all study subjects including premature and very low birth weight infants, while one study reported a proportion of failed scans. Quantitative US appeared well-tolerated, had no adverse side effects, and was appropriate for use for both single and serial scans. Fewtrell et al. [25] reported failed scans, due to technical problems. In that study, 17 of 99 patients had at least one failed scan and 4 patients had no successful scans at all. There were no clinical features or patterns related to the failed scans, but it was suggested that oedema from illness or fat deposition from rapidly growing infants could be affecting scan success.

### Reproducibility

Reproducibility of the technique (as mentioned in 11 studies) is summarised in Table 3. Intraobserver coefficient variant, interobserver coefficient variant and instrumental precision coefficient variant were all less than 2%. Instrumental precision reported for Omnisense 7000P is 0.25–0.5%.

**Table 3:** Reproducibility of quantitative ultrasound technique

Study	Year	Equipment name/model	Number of patients	Intraobserver coefficient variant (%)	Interobserver coefficient variant (%)	Instrumental precision coefficient variant (%)	Intersite variation coefficient variant (%)
Mercy et al. [2]	2007	Omnisense 7000P	84			1.26	
McDevitt et al. [8]	2007	Omnisense 7000P	39	1.1	1.2		
Zuccotti et al. [13]	2011	Omnisense 7000P	116			0.34	
Gonnelli et al. [15]	2004	DBM Bone Profiler	140			1.0	
McDevitt et al. [20]	2005	Omnisense 7000P	110	1.2			2.4
Rack et al. [23]	2012	Osteon KIV	172			0.62	
Fewtrell et al. [25]	2008		99	1–2			
Littner et al. [31]	2003	Omnisense 7000P	73	<1.2			
Rubinacci et al. [32]	2003	DBM Sonic 1200	94			1.76 (standardised)	
Littner et al. [34]	2005	Omnisense 7000P	22	<1.2			
Liao et al. [38]	2010	Omnisense 7000P	267	1.23–1.84			

No significant differences were found in readings taken from different anatomical sites [2]. The ability to take measurements from various sites has significant potential advantages and the absence of large differential measurement errors between sites is important.

### Quantitative US values

Table 1 summarises the equipment used and speed of sound values in the 29 reviewed studies. Most studies (23) used Omnisense 7000P at the tibial site, and their values were comparable for the term and preterm populations.

### Speed of sound and gestational age

Regardless of quantitative US equipment used, a positive correlation was found between speed of sound values and gestational age, with term infants having higher speed of sound values than preterm infants reflecting the increased maturity of their bones. It is to be noted that significant correlation does not mean diagnostic accuracy in any of the presented results.

Ashmeade et al. [7] found a positive correlation between speed of sound and gestational age in preterm but not in term infants. Similarly, Zuccotti et al. [13] found no correlation between gestational age and speed of sound values in term infants. Conversely, Tansug et al. [14] suggested that speed of sound and gestational age are positively correlated when reviewing values from preterm and term infants as a whole, but the correlation did not seem to apply to the preterm group alone. The small sample size (three infants with gestational age <28 weeks) could be the reason for this finding.

### Postnatal trend of speed of sound values

Postnatal speed of sound values decrease in preterm infants. A similar decrease has been seen in term infants [15–17]. This is mentioned in 14 studies and summarised in Table 4. As postnatal age increases, speed of sound values decrease despite overall growth, as shown by limb length and biochemical markers [18]. The rate of decline in speed of sound values is related to the prematurity of the infant, with most preterm infants having the steepest decline in speed of sound values [7, 17, 19]. This trend seems counterintuitive as one would expect bone density and strength to increase as infants grow. This may be because the postnatal trend of speed of sound values in preterm infants differs from that of term infants, and quantitative US is able to reflect a decline in either quantitative or qualitative bone properties despite linear growth.

**Table 4:** Postnatal trend in quantitative ultrasonography values

Reference	Year	Quantitative ultrasound device	Site/parameter	Trend of speed of sound/bone transmission time values postnatally (preterm)	Trend of speed of sound/ bone transmission time values postnatally (term)	Comments
Mercy et al. [2]	2007	Omnisense 7000P	Tibia/SOS	Decreasing		The overall trend in tibial SOS showed a decrease with postnatal age.
Ashmeade et al. [7]	2007	Omnisense 7000P	Tibia/SOS	Decreasing		There was a significant decrease over time for entire cohort of preterm infants.
Tansug et al. [14]	2011	Omnisense 7000P	Tibia/ SOS	Decreasing		SOS values of preterm infants decreases until 2nd month of life.
Gonnelli et al. [15]	2004	DBM Sonic	Humerus/BTT, SOS		Decreasing in SOS Increasing in BTT	Decrease in SOS values for term infants at 12-months follow-up. Steady increases in BTT for term infants after birth at 12-months follow up.
Betto et al. [16]	2014	DBM Sonic	Metacarpal/BTT, SOS	Decreasing	Decreasing	Deflection of metacarpal BTT from birth to 3rd week of life, followed by increase in this parameter during first few months of life.
Ritschl et al. [17]	2005	DBM Sonic	Second metacarpal/ BTT, SOS	Decreasing in SOS Increasing in metacarpal BTT	Decreasing in SOS	Decline in SOS values for up to 6 months in term and preterm infants, then increasing trend up to 18 months of life. Steady increase in metacarpal BTT after birth in preterm infants.
Litmanovitz et al. [18]	2007	Omnisense 7000P	Tibia/ SOS	Decreasing		Bone SOS decreases during the first 4 postnatal weeks in very low birth weight premature infants.
Liao et al. [19]	2005	Omnisense 7000P	Tibia/SOS	Decreasing		The SOS of infants showed an inverse correlation with postnatal age, and the decrease of bone SOS with age in premature infants was more marked than in full-term infants.
Altuncu et al. [21]	2007	Omnisense 7000P	Tibia/SOS	Decreasing		Serial assessment of tibia SOS z-scores of preterm infants showed that tibia SOS z-scores of preterm infants at term-CA (corrected age) were significantly lower than the scores at first postnatal week of life.
Rack et al. [23]	2012	Osteoson KIV	4 different sites/SOS	Decreasing		Rapid decline in SOS values in first few weeks of life, plateauing after 40 weeks post-conceptual age.
Fewtrell et al. [25]	2008	Omnisense 7000P	Tibia/SOS	Decreasing		Both absolute and z-scores relative to cross-sectional reference data fell during the postnatal period.
Litmanovitz et al. [29]	2003	Omnisense 7000P	Tibia/SOS	Decreasing		
Rubinacci et al. [32]	2003	DBM Sonic	Humerus/BTT, SOS	Decreasing		
Savino et al. [39]	2013	DBM sonic	Metacarpal/BTT, SOS	Decreasing		Decreasing trend of SOS values lasted up to 240 days, followed by slow increases in next months.

*BTT* bone transmission time, *SOS* speed of sound

### Catch-up growth

Catch-up growth of preterm infants has been documented from longitudinal studies. This is shown by the postnatal equalising of speed of sound values between preterm and term infants. McDevitt et al. [8] reported that catch-up in speed of sound values is independent of postnatal growth and occurs in most infants by 6 months. The fastest rate of catch-up in speed of sound values was seen in infants who had the lowest initial speed of sound. This finding agrees with Tansug et al. [14], who demonstrated no significant difference in speed of sound values between term and preterm infants by month 12. A similar catch-up phenomenon was seen for metacarpal bone transition time in the preterm cohort in Ritschl et al. [17]. In this study, metacarpal bone transmission time values were stable for the term cohort, and the preterm cohort displayed increasing metacarpal bone transmission time values after birth, reaching the values of term infants at around 6 months of life [17].

### Anthropometry

There are contradicting reports on whether speed of sound values are positively correlated, negatively correlated or not significantly correlated to birth weight. This is evaluated in 19 studies and summarised in Table 5. In Tansug et al. [14], Day 10 speed of sound values correlated with birth weight when considering both preterm and term infants as a whole, but when looking at preterm infants alone, there was no significant correlation. However, as previously alluded to, a limitation is the small number of preterm births included in this study. Zuccotti et al. [13] only looked at term infants and found no relation between weight and speed of sound values. In Ashmeade et al. [7], there was a significant positive correlation between speed of sound measurements and birth weight among preterm infants. In contrast, the correlation was negative in term infants. This suggests that lower rates of intrauterine growth are associated with high speed of sound values at birth.

**Table 5:** Correlation between birth weight and quantitative ultrasonography (US) values

Reference	Year	Quantitative US device	Site/parameter	Correlation between birth weight and quantitative US values		Comments
				Preterm infants	Term infants	
Mercy et al. [2]	2007	Omnisense 7000P	Tibia/SOS	Positive correlation		Significant positive correlation between birth weight and SOS values when using first measure cross-sectional data.
Ashmeade et al. [7]	2007	Omnisense 7000P	Tibia/SOS	Positive correlation	Negative correlation	Significant positive correlation in birth weight and SOS measurements in preterm infants, but negative correlation in term infants. This might suggest that lower rates of interuterine growth are associated with high SOS values.
McDevitt et al. [8]	2007	Omnisense 7000P	Tibia/SOS	No significant correlation		No significant effect of weight or length gain on SOS values.
Zuccotti et al. [13]	2011	Omnisense 7000P	Tibia/SOS		No significant correlation	No relation between birth weight and SOS values.
Tansug et al. [14]	2011	Omnisense 7000P	Tibia/SOS	No significant correlation		There is positive correlation between birth weight when considering preterm and term infants as a whole, but no significant correlation when looking at preterm infants alone. There are only a small number of preterm births included in this study.
Gonnelli et al. [15]	2004	DBM Bone profiler	Humerus/BTT, SOS		Positive correlation	BTT and humerus BTT of neonates showed significant relationship with birth weight.
Betto et al. [16]	2014	DBM Sonic	Metacarpal/BTT, SOS	Positive correlation		Weight and length at 3rd week and 36th week of life correlated positively with metacarpal BTT.
Ritschl et al. [17]	2005	DBM Sonic	Second metacarpus/ BTT, SOS	Positive correlation	Positive correlation	Quantitative US parameters were closely correlated with length and weight of infant.
Liao et al. [19]	2005	Omnisense 7000P	Tibia/SOS	No significant correlation	No significant correlation	SOS in infants with birth weights <1,500 g was lower than in infants with birth weights >2,500 g. However, there are no significant differences after accounting for gestational age and birth season.
McDevitt et al. [20]	2005	Omnisense 7000P	Tibia, distal third of radius/SOS	32–36 weeks’ gestational age: no significant correlation <32 weeks’ gestational age: negative correlation	No significant correlation	There was no significant difference in SOS for SGA and AGA infants in >37 weeks’ gestational age and 32–36 weeks’ gestational age groups. In the <32 weeks’ gestational age group, SGA infants had higher SOS values than AGA infants. However, there was no significant difference between LGA and AGA infants in all groups.
Chen et al. [22]	2012	Omnisense 7000P	Tibia/SOS	Negative correlation	Negative correlation	Birth weight had a negative effect on increasing SOS values. SOS values were higher in SGA infants than in AGA infants.
Rack et al. [23]	2012	Osteoson KIV	4 different sites/SOS	Positive correlation	No significant correlation	Birth weight was the strongest predictor of quantitative US values in the most immature infants, but predictive value becomes insignificant in term infants.
Fewtrell et al. [25]	2008	Omnisense 7000P	Tibia/SOS	No significant correlation		There is no significant correlation between SOS and birth weight at time of scan.
Littner et al. [31]	2003	Omnisense 7000P	Tibia/SOS	Positive correlation	Positive correlation	SOS values were more closely correlated to gestational age than with birth weight.
Rubinacci et al. [32]	2003	DBM Sonic	Humerus/BTT, SOS	Positive correlation		SOS values were found to be significantly correlated to birth weight and weight at measurement (postconceptual age of at least 34 weeks for preterm infants).
Littner et al. [33]	2004	Omnisense 7000P	Tibia/SOS		Negative correlation	LGA infants had lower SOS values than normal AGA values predicted from standard curves.
Teitelbaum et al. [35]	2006	Omnisense 7000P	Tibia/SOS	Positive correlation	Positive correlation	There was a significant positive correlation between SOS and birth weight, independent of gestational age.
Liao et al. [38]	2010	Omnisense 7000P	Tibia/ SOS	Positive correlation	Positive correlation	SOS values of infants with birth weight of <1,500 g was significantly lower than infants with birth weight of >2,500 g.
Savino et al. [39]	2013	DBM Sonic	Metacarpal/ BTT, SOS	No significant correlation	No significant correlation	Negative correlation was observed between SOS, length and weight. However with multiple regression modelling, no significant relationship was found.

*AGA* appropriate for gestational age, *BTT* bone transmission time, *LGA* large for gestational age, *SGA* small for gestational age, *SOS* speed of sound

Perhaps more interesting is the new insight into appropriate, small and large for gestational age infants and how their speed of sound values differ. Ten studies in this review have made mention of the effects of size for gestational age on speed of sound values (Table 6).

**Table 6:** Relationship between speed of sound values of appropriate for gestational age (AGA), small for gestational age (SGA) and large for gestational age (LGA) infants

Study	Year	Quantitative ultrasonography device	Site/parameter	Relationship between speed of sound values of AGA and SGA infants	Relationship between speed of sound values of AGA and LGA infants
Mercy et al. [2]	2007	Omnisense 7000P	Tibia/ SOS	Rapid decline in SOS values in SGA infants postnatally as compared to AGA infants.	
Ashmeade et al. [7]	2007	Omnisense 7000P	Tibia/ SOS	SOS values were higher in SGA infants as compared to AGA infants.	
Liao et al. [19]	2005	Omnisense 7000P	Tibia/SOS	No difference in SOS values between SGA and AGA infants.	No difference in SOS values between AGA and LGA infants.
McDevitt et al. [20]	2005	Omnisense 7000P	Tibia, distal third of radius/ SOS	>32 weeks’ gestation: No significant difference in SOS values between AGA and SGA infants <32 weeks’ gestation: SGA infants had higher SOS values than AGA infants	
Altuncu et al. [21]	2007	Omnisense 7000P	Tibia/SOS	No difference in SOS values between SGA and AGA infants.	
Chen et al. [22]	2012	Omnisense 7000P	Tibia/ SOS	SOS values were higher in SGA infants with higher gestational age as compared to AGA infants with similar birthweight.	
Rack et al. [23]	2012	Osteoson KIV	4 different sites/ SOS	Lower SOS values in SGA infants than AGA infants.	
Littner et al. [24]	2004	Omnisense 7000P	Tibia/SOS		LGA infants were found to have lower SOS values than AGA infants.
Littner et al. [34]	2005	Omnisense 7000P	Tibia/SOS	SGA infants have higher SOS values than AGA controls.	
Chen et al. [36]	2007	Omnisense 7000P	Tibia/SOS	Preterm SGA infants had higher tibial SOS values than their AGA counterparts; findings were similar regardless of the reference chart used to categorize infants as SGA or AGA.	

*SOS* speed of sound, *US* ultrasonography

McDevitt et al. [20] found no significant difference in speed of sound values between small for gestational age and appropriate for gestational age infants of more than 32 weeks’ gestation. Younger than 32 weeks’ gestation, small for gestational age infants had higher speed of sound values than appropriate for gestational age infants. Liao et al. [19] and Altuncu et al. [21] also found no difference in speed of sound values between small for gestational age and appropriate for gestational age infants. Chen et al. [22] suggested that the higher speed of sound may be attributable to the older gestational age in small for gestational age infants compared to appropriate for gestational age infants with similar birth weight. This may show that maturity of the fetus has a larger bearing on bone speed of sound than birth weight. However, Rack et al. [23] reported lower speed of sound values in small for gestational age infants than appropriate for gestational age infants. This could be explained by a deficiency in calcium and phosphate leading to reduced placental transfer and diminished bone mineralisation in small for gestational age infants or perhaps a soft-tissue effect causing higher speed of sound values in small for gestational age infants than appropriate for gestational age infants. Mercy et al. [2] found a rapid decline in speed of sound values postnatally in small for gestational age infants as compared to appropriate for gestational age infants, while there was an upward trend for large for gestational age infants. There were no explanations provided, but it was stated that this is the first time such a trend has been reported.

In Littner et al. [24], large for gestational age infants were found to have lower speed of sound values than appropriate for gestational age infants. This finding is not reproduced in Liao et al. [19], where it was concluded that no differences in speed of sound values were found between appropriate for gestational age, small for gestational age and large for gestational age infants. Littner et al. [24] speculate that the relative lack of motion of macrosomic infants as compared to appropriate for gestational age infants may lead to lower speed of sound, as physical activity is known to enhance mineral accretion.

### Biochemical bone markers

Fewtrell et al. [25], Chen et al. [26] and Tansug et al. [14] did not find any relationship between speed of sound values and the bone turnover markers serum alkaline phosphatase and serum phosphate. In Chen et al. [26], there was only a slight upward trend in alkaline phosphatase, which did not correlate with any speed of sound trends. Serum alkaline phosphatase is the sum of three isoforms from the liver, intestines and bone, as such an increase in serum alkaline phosphatase might be due to a liver dysfunction. Tansug et al. [14] explained that their findings might be because there were no infants with very low serum phosphate or high serum alkaline phosphatase in their study. As a high serum alkaline phosphatase is known to develop relatively late in the pathological process of metabolic bone disease, Fewtrell et al. [25] aimed to assess the ability of early speed of sound measurements to predict a high serum alkaline phosphatase level later on. They found that speed of sound measurements did not predict a high alkaline phosphatase. Conversely, a high serum alkaline phosphatase was also not associated with a lower final speed of sound measurement. However, this study did not consider some confounding factors, such as factors related to the severity of illness or infant characteristics such as gestational age or birth weight. Conversely, Altuncu et al. [21] found that there was an inverse correlation between alkaline phosphatase levels and tibia z score at term corrected age in preterm infants. In their study, patients with alkaline phosphatase>900 international units per litre were found to have significantly lower tibia z score for speed of sound, indicating ongoing osteoblastic activity [21].

Other studies have found significant correlations between biochemical markers and speed of sound values. McDevitt et al. [8] found that serum phosphate and speed of sound were significantly positively correlated. This correlation is replicated in Betto et al. [16], with another quantitative US parameter. The study found that metacarpal bone transmission time was correlated to serum phosphate, phosphaturia and calciuria in the third week of life and suggested that these three biochemical tests could be used in the workup of metabolic bone disease. This observation was also made in Ashmeade et al. [7] and Rack et al. [23]. Additionally, in Ashmeade et al. [7], a significant negative correlation was found at various time points between serum alkaline phosphatase and speed of sound values. This shows that serum markers in combination with longitudinal speed of sound measurements may be useful for identifying infants at risk of developing metabolic bone disease. Rack et al. [23] also found a negative correlation between serum alkaline phosphatase and quantitative US parameters. The study also measured urine calcium and phosphate concentrations and serum calcium concentration and found that none of these variables correlated with quantitative US, contrary to Betto et al. [16].

Litmanovitz et al. [18] used bone specific alkaline phosphatase and carboxy terminal cross-links telopeptide of Type-I collagen as markers of bone formation and bone resorption, respectively. They found that although there was a significant increase in bone specific alkaline phosphatase and significant decrease in carboxy terminal cross-links telopeptide of Type-1 collagen, both parameters remained within the normal range and there were no significant correlations between bone turnover markers and speed of sound.

## Summary of findings

In neonates, quantitative US can be measured with Omnisense 7000P, DBM sonic and Osteon KIV devices. The measurements are well tolerated by all infants, even those in intensive care. This review did not compare the reliability of different US devices; however, the trend of speed of sound values was similar for each device. Intraobserver, interobserver and intersite precision were high in all devices. The studies reviewed showed a difference between preterm and term infants at birth, and a decreasing trend in speed of sound values in preterm infants when longitudinal measurements were taken. This may reflect either that the postnatal trend of speed of sound values in preterm infants differs from term infants, or that quantitative US is able to assess both quantitative and qualitative bone properties, and gives a more holistic picture of bone health. Catch-up growth of preterm infants has been demonstrated in longitudinal studies.

Although quantitative US is now widely used in adults in the context of osteoporosis, its use in infants and children is limited to studies of small sample size [23]. Lack of reference data, use of different quantitative US devices and assessment of different sites makes it challenging to compare the outcome between studies [27]. The correlation of quantitative US parameters with various factors mentioned in this review, for example biochemical markers and anthropometry, has not provided consistent results. The correlation between quantitative US parameters and the current gold standard assessment DXA is also lacking consistent data [22]. US reference values are available for term and preterm infants, but they are specific to the manufacturer of the device used and standardised values have not been achieved [28]. Most importantly, values for predicting or monitoring metabolic bone disease have not been established [14].

## Conclusion

The noninvasive, financially viable and convenient monitoring of bone health with US might hold potential as an initial screening tool to predict metabolic bone disease but also for follow-up to review treatment efficacy and assess subsequent trends in bone health. However, the results presented in the papers we evaluated were not always concordant. More studies focusing on the association of biochemical bone markers, DXA, radiographs and quantitative US parameters will be essential in assessing the accuracy and reproducibility of quantitative US variables before widespread clinical use on neonatal units.

## Electronic supplementary material

ESM 1 (DOC 42 kb)
