# Supplementary material for: Feasibility of quantitative ultrasonography for the detection of metabolic bone disease in preterm infants — systematic review
Source: Pediatr Radiol. 2018 Jun 16;48(11):1537–49. doi: 10.1007/s00247-018-4161-5 (PMC6153869; doi:10.1007/s00247-018-4161-5)
Supplement: Supplementary file 1 — (DOC 42 kb) [file 247_2018_4161_MOESM1_ESM.doc]

**Supplementary table 1** Results of database searches

| **Database** | **Search term/result code** | **Combined with (OR)** | **Combined with (AND)** | **Limited to** | **Results**  *n* **(code)** |
| --- | --- | --- | --- | --- | --- |
| EMBASE | Bone disease of prematurity |  |  |  | 45 (**A**) |
|  | Expand METABOLIC BONE DISEASE (141951) | OSTEOPENIA (16066)  BONE DENSITY (73677)  BONE EXAMINATION/BONE (239143)  BONE STRENGTH (9649)  Bone health (6985)  Bone status (1204)  Bone mineral status (393)  Bone mineral density (43807)  Bone quality (5085) |  |  | 378197 (**B**) |
|  | **B** |  |  | Infant to 1 year | 1657 (**C**) |
|  |  | Infant*  Preterm*  Newborn* |  |  | 528347 (**D**) |
|  | **B** |  | **D** |  | 6480 (**E**) |
|  | Osteopenia (12047) |  | Prematur* (150686) |  | 339 (**F**) |
|  | ULTRASOUND (198318) | Qus (1560)  Ultrasound or ultrasonography* (362951) Ultrasono* (118369) |  |  | 413042 (**G**) |
|  | **C** |  | **G** | Infant to 1 year | 90 |
|  | **E** |  | **G** |  | 358 |
|  | **A** | **F** |  |  | 377 (**H**) |
|  | **G** |  | **H** |  | 24 |
|  | 90,358,96 duplicates removed |  |  |  | 385 |
| Medline | Bone disease of prematurity |  |  |  | 31 (**I**) |
|  | Expand BONE DISEASES, METABOLIC (67514) | Bone health (4327)  Bone status (857)  Bone mineral status (306)  Bone mineral density (31258)  Bone quality (3716)  Osteopenia (7700)  BONE DENSITY (44721)  BONE AND BONES (79682)  Bone strength (3888) |  |  | 168282 (**J**) |
|  | **J** |  |  | Infant,newborn  Infant | 7464 (**K**) |
|  |  | Infant*  Preterm*  Newborn* |  |  | 449579 (**L**) |
|  | **J** |  | **L** |  | 2978 (**M**) |
|  | osteopenia (7700) |  | Prematur* (117343) |  | 206 (**N**) |
|  | Qus (1071) | Qus ((1071)  Ultrasono* (2011)  Ultrasound or ultrasonography* (252163) |  |  | 253166 (**O**) |
|  | **K** |  | **O** | Infant,newborn  Infant | 175 |
|  | **M** |  | **O** |  | 119 |
|  | **I** | **N** |  |  | 232 (**P**) |
|  | **O** |  | **P** |  | 18 |
|  | 18,175,119 duplicates removed |  |  |  | 200 |
